# Relation of Circulating Resistin to Insulin Resistance in Type 2 Diabetes and Obesity: A Systematic Review and Meta-Analysis

**DOI:** 10.3389/fphys.2019.01399

**Published:** 2019-11-19

**Authors:** Kai-zhen Su, Yan-run Li, Di Zhang, Jun-hua Yuan, Cai-shun Zhang, Yuan Liu, Li-min Song, Qian Lin, Man-wen Li, Jing Dong

**Affiliations:** ^1^Clinical Medicine Department, Medical College, Qingdao University, Qingdao, China; ^2^Special Medicine Department, Medical College, Qingdao University, Qingdao, China; ^3^Physiology Department, Medical College, Qingdao University, Qingdao, China

**Keywords:** resistin, insulin resistance, relation, diabetes mellitus, obesity, hyperresistinemia

## Abstract

**Background:** Resistin, a cysteine-rich polypeptide encoded by the RETN gene, which plays an important role in many mechanisms in rodent studies, including lipid metabolism, inflammation and insulin resistance. Nevertheless, the relationship between resistin and insulin resistance in humans is under debate. The present study was designed to clarify the correlation between resistin and insulin resistance.

**Methods:** A systematic literature search was performed using PubMed, Embase and Cochrane Library until March 3, 2019 with the keywords “resistin” and “insulin resistance.” Funnel plots and Egger's test were used to detect publication bias. A random-effects model was used to calculate the pooled effect size. Subgroup analysis and meta regression was performed to identify the sources of heterogeneity.

**Results:** Fifteen studies were included in our systematic review. Among them, 10 studies with Pearson coefficients were used for meta-analysis. We found resistin levels were weakly correlated with insulin resistance in those with T2DM and obesity (*r* = 0.21, 95% CI: 0.06–0.35, *I*^2^ = 59.7%, *P* = 0.003). Nevertheless, subgroup analysis suggested that circulating resistin levels were significantly positively correlated with insulin resistance in individuals with hyperresistinemia (≥14.8 ng/ml) (*r* = 0.52, 95% CI: 0.35–0.68, *I*^2^ = 0.0%, *P* = 0.513). And there was no relationship between circulating resistin and insulin resistance in those with normal circulating resistin levels (<14.8 ng/ml) (*r* = 0.08, 95% CI: −0.01–0.18, *I*^2^ = 0.0%, *P* = 0.455). Publication bias was insignificant (Egger's test *P* = 0.592).

**Conclusion:** In T2DM and obese individuals, resistin levels were positively correlated with insulin resistance in those with hyperresistinemia, but not in those with normal circulating resistin levels.

## Introduction

Type 2 diabetes, characterized by insulin resistance (IR), is a complex chronic disorder of which the prevalence increased markedly in recent years (DeFronzo et al., 2015). In 2015, 415 million people were estimated to have diabetes, more than 90% of whom had type 2 diabetes (GBD Disease Injury Incidence Prevalence Collaborators, 2016). Obesity currently affects more than 600 million people worldwide by recent estimates and it was also characterized by defects of insulin action (Lois and Kumar, 2009). As the common pathology in obesity and type 2 diabetes, IR is defined as a state in which cells fail to respond to insulin, resulting in the development of hyperglycemia (Sah et al., 2016). In clinical studies, concerned about the problem of compliance with hyperinsulinemic euglycemic clamps, HOMA (homeostasis model assessment) has been applied more widely to estimate insulin sensitivity (Stern et al., 2005).

Resistin, also known as FIZZ3 or adipose tissue-specific secretory factor (ADSF), is a cysteine-rich polypeptide encoded by the RETN gene (Wang et al., 2002). The hormone was first discovered in a screen for targets of thiazolidinediones (TZDs) in white adipose of mice; it is believed to play a significant role in the development of insulin resistance (Steppan et al., 2001). The structure and distribution of resistin in humans are quite different from that of rodents. Murine resistin is a 114-amino acid polypeptide primarily produced in adipose tissues (Muse et al., 2004). While in humans, it is proved to be a 108-amino acid polypeptide expressed in adipocytes, pancreatic cells, muscle, and mononuclear cells (Dietze et al., 2002; Minn et al., 2003; Patel et al., 2003). It is worth mentioning that peripheral blood mononuclear cells (PBMCs) are key producers of resistin in humans (Kusminski et al., 2005). Its role in pro-inflammatory processes has been demonstrated in several studies (Kaser et al., 2003; Bokarewa et al., 2005).

Consistent with their varying structures, several functions of resistin vary between human and rodents. Rodent studies showed a clear role of resistin in the development of insulin resistance. Transgenic overexpression or recombinant of resistin impaired insulin function, while administration of anti-resistin antibody and antisense oligonucleotide treatment improved insulin sensitivity in mice (Steppan et al., 2001). However, the role of resistin in insulin resistance remains controversial in humans. Several studies found positive correlations between resistin and insulin resistance in T2DM, obese and healthy individuals (Al-Harithy and Al-Ghamdi, 2005; Hivert et al., 2008; Zaidi and Shirwany, 2015); this discovery appears to be supported by studies reporting significantly higher resistin levels in populations with T2DM (Heilbronn et al., 2004). Nevertheless, a substantial number of studies have failed to find correlations between resistin and insulin resistance (Gerber et al., 2005; Bu et al., 2012).

To date, no systematic reviews or meta-analysis has analyzed the association between resistin and insulin resistance in populations; therefore, we analyzed this association in the present study.

## Methods

### Search Strategy

The following databases were searched by two of the authors independently until March 3, 2019: PubMed, Cochrane Library and Embase. Two groups of keywords and their MeSH terms searched in PubMed were used to locate the relevant studies: resistin (e.g., “adipocyte secreted factor,” “adipocyte specific secreted factor,” “adipose tissue specific secreted factor,” “ADSF,” “FIZZ3,” “FIZZ3 protein,” “found in inflammatory zone 3 protein,” “protein FIZZ3,” “Adipocyte Cysteine-Rich Secreted Protein FIZZ3,” “Adipocyte Cysteine Rich Secreted Protein FIZZ3”) and insulin resistance (e.g., “Resistance, Insulin,” “Insulin Sensitivity,” “Sensitivity, Insulin,” “resistance, insulin”). In order to make sure that there were not any articles missed, we did not limit the starting years. In addition, manual searches of the references of relevant studies were also conducted.

### Inclusion and Exclusion Criteria

The inclusion criteria were set as follows: (1) observational study or baseline data of an experimental study; (2) participants aged ≥18 years with simple obesity, simple T2DM or both; (3) presence of correlation coefficients regarding resistin and insulin resistance measured using the homeostasis model assessment-insulin resistance (HOMA-IR); (4) Pearson's or Spearman's tests used to perform the correlation analyses; (5) publication in English language. And the studies were excluded if they met the following criteria: (1) reviews, comments, protocols, meeting abstracts, case report, or letters; (2) the participants of the study had other disorders, such as metabolic syndrome, non-alcoholic fatty liver disease or polycystic ovary syndrome.

### Data Extraction and Quality Assessment

For data extraction, two of the authors reviewed the titles and abstracts of articles and then screened the full texts independently. The data were collected after all disagreements were resolved. We extracted the basic information (first author, year of publication, sample size, country) and participant characteristics (age, gender, BMI, fasting blood glucose, resistin, HOMA-IR, insulin) from the articles. And the data were converted to uniform units as needed. The Pearson's correlation coefficient was also extracted to obtain the effect size. In addition, we collected the Spearman's correlation coefficients to perform the systematic review.

The quality assessment was conducted using NOS (Newcastle-Ottawa Scale), including eight items, respectively (four items for selection, one item for comparability, three items for outcome in the scale of cross sectional studies and four items for selection, one item for comparability, three items for exposure in the scale of case control studies); NOS was used for cross-sectional studies and case control studies (Stang, 2010). A study was awarded stars according to the requirement of various questions. To assess the quality of the studies, study scores (ranging from 0 to 9 for case control studies) were classified as “high quality” (scores ≥ 6) or “low quality” (scores < 6) for further analysis.

We also used the Cochrane Collaborations tool to assess the reporting quality in randomized clinical trials (Higgins et al., 2011). The list of items in the tool includes seven items: random sequence generation, allocation concealment, blinding of participants and personnel, blinding of outcome assessment, incomplete outcome data, selective reporting and other bias. Two authors performed the assessment independently according to the tool protocol.

### Statistical Analysis

Pearson correlation coefficients were transformed into Fisher's *z* values to calculate the variance and corresponding data, and then we conducted meta-analysis and calculated the 95% confidence intervals (CI). Heterogeneity between studies was tested via *I*^2^ tests and Cochran's *Q* tests. Studies which met the criteria of *I*^2^ > 50% in *I*^2^ tests and *p* < 0.10 in Cochran's *Q* tests simultaneously were defined as having significant heterogeneity. If *I*^2^ < 50% or *p* > 0.10, studies were considered as having low heterogeneity. We adopted a random-effects model when there was significant heterogeneity and publication bias was evaluated using visual assessment of funnel plot asymmetry and Egger's tests. In addition, subgroup analyses, meta-regression and sensitivity analyses were performed. We conducted all these statistical analyses using Stata software version 12.0.

## Results

### Search Results and Study Inclusion

**Figure 1:**
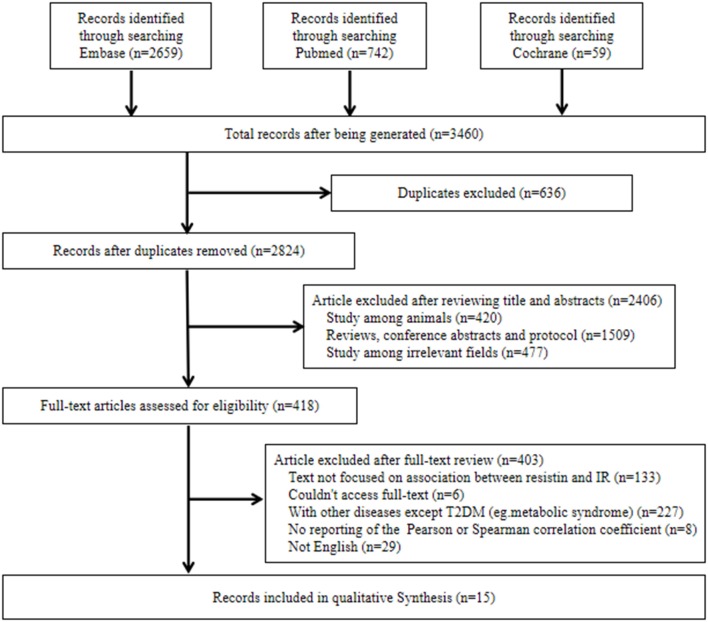
Flow chart of literature search. IR, insulin resistance; T2DM, type 2 diabetes mellitus.

Initially, the literature search identified 3,460 potential studies. After duplication checking, review of titles, abstracts and full texts, 15 eligible studies were included in this systematic review. Among them, 10 studies reported the correlations between resistin levels and IR with Pearson coefficients were used for further meta-analysis. The flow diagram of the search procedure is presented in Figure 1.

### Study Characteristics

In the studies of Zaidi et al., Jung et al., Stepien et al., and Kaplon-Cieslicka et al., patients were divided into two groups according to their study designs, and the correlation coefficients were given, respectively (Jung et al., 2005; Stepien et al., 2014; Kaplon-Cieslicka et al., 2015; Zaidi and Shirwany, 2015). Therefore, we included 15 studies with 20 groups, a total of 1,227 patients. Seven of the groups were simple obesity (Owecki et al., 2011; Stepien et al., 2011, 2014; Zaidi and Shirwany, 2015; Parreno Caparros et al., 2017), ten of them were simple T2DM (Al-Harithy and Al-Ghamdi, 2005; Jung et al., 2005; Park et al., 2006; Mojiminiyi and Abdella, 2007; Tokuyama et al., 2007; Gharibeh et al., 2010; Bu et al., 2012; Yin et al., 2012; Kaplon-Cieslicka et al., 2015), two of them were obesity with T2DM (Mabrouk et al., 2013; Zaidi and Shirwany, 2015), and one of them was T2DM with or without obesity. The publication years ranged from 2005 to 2017, and the sample sizes ranged from 13 to 140. Fasting serum or plasma resistin levels were measured using enzyme linked immunosorbent assay (ELISA). More details of the included studies are displayed in Table 1.

**Table 1:** Characteristics of included studies.

**Author**	**Year**	**Country**	**Study design**	**Study population**	**Number (male/** **female)**	**Age**	**BMI**	**Blood glucose**	**Insulin**	**HOMA-IR**	**Resistin**	**Correlation coefficient between resistin** **and IR**	**Correlation coefficient type**	**Blood sample**
Al-Harithy and Al-Ghamdi (2005)	2005	Saudi	Cross sectional	44	0/44	45 ± 10	25.5 ± 4.9	8.74 ± 4.04 mmol/l	12.96 ± 5.15 μIU/mL	5.38 ± 6.63	19.42 ± 3.60 ng/ml	0.49	Pearson	Serum
Jung et al. (2005)	2005	Korean	RCT^d^	14	6/8	60 ± 8	23.3 ± 2.6	11.4 ± 2.1 mmol/l	85 ± 39 pmol/l	7.3 ± 3.3	2.49 ± 1.93 ng/ml	0.3	Pearson	Plasma
Jung et al. (2005)	2005	Korean	RCT^d^	13	6/7	54 ± 14	24.6 ± 2.4	10.3 ± 2.7 mmol/l	68 ± 20 pmol/l	5.3 ± 2.4	2.61 ± 1.69 ng/ml	0.1	Pearson	Plasma
Park et al. (2006)	2006	Japan	Cross sectional	104	53/51	53.8 ± 9.2	23.87^c^	≥7.0 mmol/l	–	2.241^c^	6.527 ng/ml^c^	0.157	Pearson	Serum
Mojiminiyi and Abdella (2007)	2007	Kuwaiti	Cross sectional	135	57/78	58.92^c^	31.21^c^	9.928 mmol/l^c^	21.239 μIU/mL^c^	8.964^c^	23.61 ng/ml^c^	0.3	Spearman	Plasma
Tokuyama et al. (2007)	2007	Japan	Cross sectional	113	77/36	55.8 ± 13.0	24.7 ± 3.88	149.1 ± 44.1 mg/dl	–	3.82 ± 5.16	10.35 ± 6.30 ng/ml	0.302	Spearman	Serum
Gharibeh et al. (2010)	2010	Jordan	Case control	140	63/77	54.36 ± 0.88	30.89 ± 0.47	10.10 ± 0.32 mmol/l	11.94 ± 0.60 μIU/mL	5.46 ± 0.37	7.82 ± 0.29 ng/ml	0.08505	Pearson	Plasma
Owecki et al. (2011)	2011	Poland	Cross sectional	136	61/75	48.8 ± 13.4	37.69 ± 7.22	5.49 ± 0.63 mmol/l	40.62 ± 54.04 μIU/mL	–	24.89 ± 9.73 ng/ml	−0.1608	Spearman	Serum
Stepien et al. (2011)	2011	Poland	Cross sectional	19	4/15	53.0 ± 13.19	33.19 ± 3.13	5.46 ± 0.50 mmol/l	6.10 ± 1.13 μIU/mL	1.47 ± 0.27	0.93 ± 0.42 ng/ml	−0.2000	Spearman	Serum
Stepien et al. (2011)	2011	Poland	Cross sectional	18	7/11	49.56 ± 14.16	34.77 ± 3.52	5.72 ± 0.52 mmol/l	14.29 ± 4.68 μIU/mL	3.64 ± 1.28	1.04 ± 0.44 ng/ml	0.1662	Spearman	Serum
Bu et al. (2012)	2012	China	Cross sectional	22	10/12	49.64^c^	22.75^c^	7.736 mmol/l^c^	7.345 μIU/mL^c^	2.013^c^	1.717 ng/ml^c^	−0.178	Pearson	Plasma
Yin et al. (2012)	2012	China	Case control	38	18/20	45 ± 10	25.5 ± 4.9	7.4 ± 1.7 mmol/l	9.2 ± 6.2 mIU/l	5.5^c^	12.3 ± 2.7 ng/ml	0.09	Pearson	Serum
Mabrouk et al. (2013)	2013	Egypt	Case control	24	8/16	32.4 ± 9	53.7 ± 5.43	161.8 ± 23.9 mg/dl	17.7 ± 1.8 μIU/mL	5.4 ± 0.65	51 ± 8.2 ng/ml	0.56	Pearson	Serum
Stepien et al. (2014)	2014	Poland	Cross sectional	16	4/12	59.3 ± 13.4^a^	34.5 ± 3.0^a^	5.1 ± 0.4 mmol/l^a^	7.5 ± 2.3 μIU/mL^a^	1.7 ± 0.5^a^	1.0 ± 0.4 ng/ml^a^	0.013	Pearson	Serum
Stepien et al. (2014)	2014	Poland	Cross sectional	48	20/28	54.8 ± 11.2	36.8 ± 4.9	5.9 ± 0.4 mmol/l	17.2 ± 8.3 μIU/mL	4.5 ± 2.3	1.0 ± 0.3 ng/ml	−0.223	Pearson	Serum
Zaidi and Shirwany (2015)	2015	Pakistan	Cross sectional	40	12/28	44 ± 7	35 ± 5	164 ± 46 mg/dl	37 ± 7 μIU/mL	19 ± 8	38 ± 8 ng/ml	0.561	Pearson	Serum
Zaidi and Shirwany (2015)	2015	Pakistan	Cross sectional	40	12/28	40 ± 6	33 ± 3	83 ± 8 mg/dl	26 ± 6 μIU/mL	5 ± 1	25 ± 5 ng/ml	0.307	Pearson	Serum
Kaplon-Cieslicka et al. (2015)	2015	Poland	Cross sectional	119	69/50	70^b^	30.4^b^	–	–	3.6^b^	7.1 ng/ml^b^	0.03	Spearman	Serum
Kaplon-Cieslicka et al. (2015)	2015	Poland	Cross sectional	76	29/47	66^b^	30.6^b^	–	–	4^b^	6.4 ng/ml^b^			Serum
Parreno Caparros et al. (2017)	2017	Spain	RCT^d^	68	22/46	39.57 ± 10.15	48.27 ± 7	114.65 ± 42.96 mg/dl	24.21 ± 15.47 mIU/l	7.39 ± 6.64	9.42 ± 3.81 ng/ml	0.201	Pearson	Plasma

Data was presented by mean ± SD.

a Data presented by mean ± SEM.

b Data presented by median.

c Data presented by mean.

d *Data was collected from the baseline*.

### Overall Meta-Analysis

**Figure 2:**
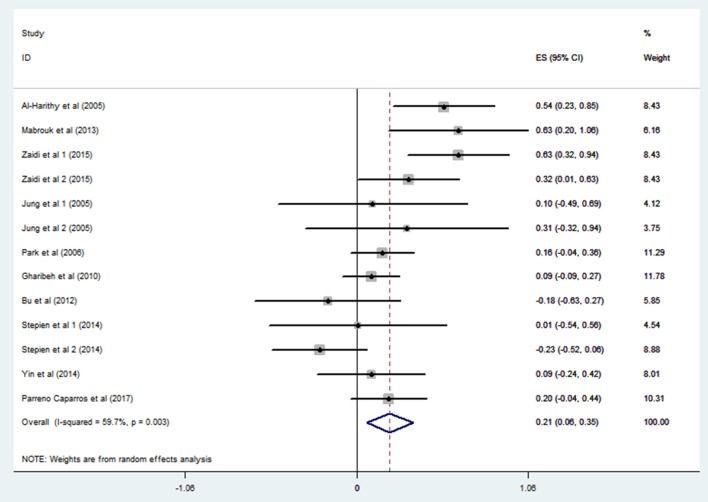
Forest plot showing the effect size of correlation coefficients between circulating resistin and insulin resistance. Summary estimates were analyzed using a random-effects model. CI, confidence interval.

**Figure 3:**
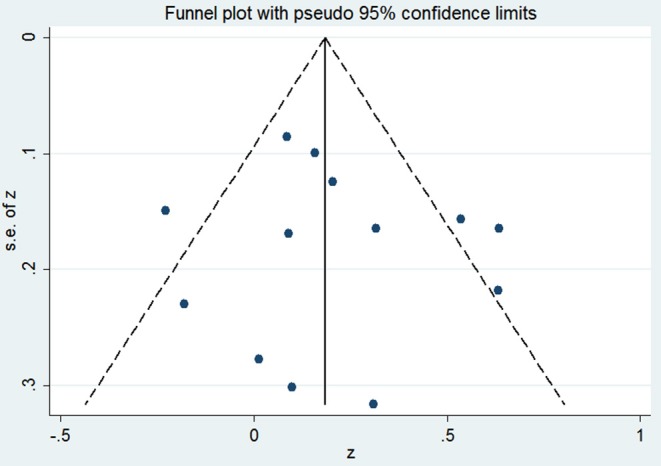
The funnel plot of the publication bias.

Ten studies including 13 groups and 611 patients with Pearson coefficients were used for meta-analysis. As demonstrated in Figure 2, resistin levels were weakly correlated with IR (*r* = 0.21, 95% CI: 0.06–0.35). The r values exhibiting significant heterogeneity were evaluated using a random-effects model (*I*^2^ = 59.7%, *P* = 0.003). No publication bias was found by inspection of funnel plots and using Egger's tests (*P* = 0.592) (Figure 3).

### Subgroup Meta-Analysis

**Figure 4:**
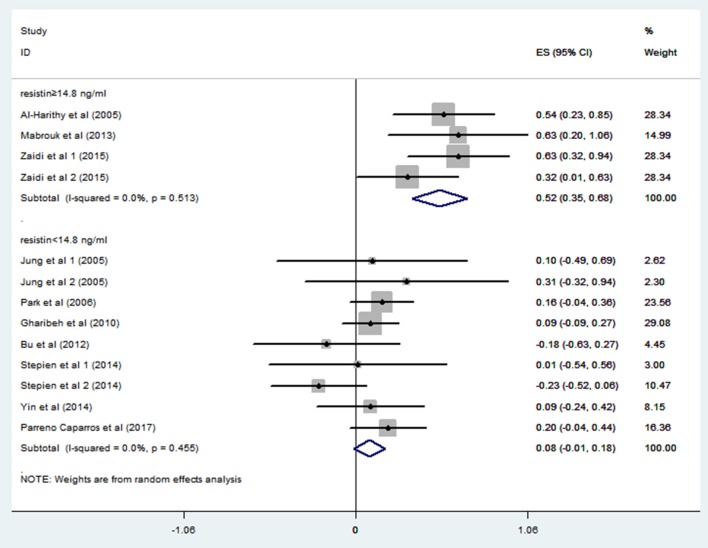
Forest plot of correlation coefficients between circulating resistin and insulin resistance based on fasting circulating resistin levels. CI, confidence interval.

In order to explore the sources of heterogeneity, we performed subgroup analysis. A study from Japan (*n* = 3192) reported quartiles of blood resistin levels in the general population (Kawamura et al., 2010). We chose the 75% point to divide these studies into a hyperresistinemia group (blood resistin ≥ 14.8 ng/ml) and a normal circulating resistin group (blood resistin < 14.8 ng/ml). In the hyperresistinemia group, resistin levels significantly positively correlated with IR (*r* = 0.52, 95% CI: 0.35–0.68, *I*^2^ = 0.0%, *P* = 0.513). However, the normal circulating resistin group did not show a significant correlation (*r* = 0.08, 95% CI: −0.01–0.18, *I*^2^ = 0.0%, *P* = 0.455) (Figure 4). After reviewing all the studies with Pearson or Spearman coefficients together, we found a similar result to this analysis. Therefore, we deduced that resistin levels were positively correlated with IR only in those with hyperresistinemia, but not in those with normal circulating resistin levels.

In the following work, we analyzed subgroups classified by Study design (RCT or cross sectional or case control) and blood sample (serum or plasma) (Table 2). However, none of these factors showed statistical significance or reduced heterogeneity in each subgroup.

**Table 2:** Subgroup analyses of correlations between insulin resistance and resistin.

	**Groups**	**Participants**	**Random effects *r* (95% CI)**	***I*^**2**^ (%)**	***P* for heterogeneity**
Overall	13	611	0.21 (0.06, 0.35)	59.7	0.003
Subgroup analysis					
**Resistin**					
Resistin ≥ 14.8 ng/ml	6	148	0.52 (0.35, 0.68)	0	0.513
Resistin < 14.8 ng/ml	7	463	0.08 (−0.01, 0.18)	0	0.455
**Study design**					
Case control	3	202	0.22 (−0.07, 0.51)	64.2	0.061
Cross sectional	7	314	0.20 (−0.05, 0.44)	74.7	0.001
RCT	3	95	0.20 (−0.01, 0.41)	0	0.892
**Blood sample**					
Serum	8	354	0.27 (0.05, 0.48)	71.7	0.001
Plasma	5	257	0.11 (−0.02, 0.23)	0	0.618

*RCT, randomized control trial; CI, confidence interval*.

### Meta-Regression

**Figure 5:**
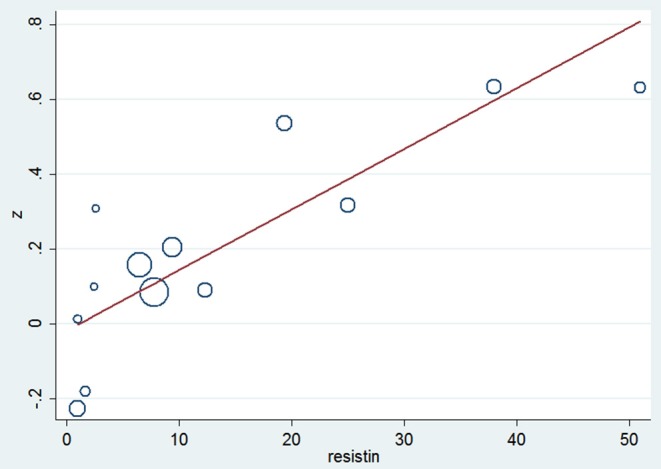
The bubble chart of the meta-regression between the correlation coefficient and resistin.

Meta-regression analysis (Table 3) indicated that resistin is a significant influence factor of the correlation coefficient (*p* = 0.001) (Figure 5). The results didn't support a relationship between the correlation coefficient and other influence factors including age, BMI, blood glucose and insulin (*p* > 0.05 for all).

**Table 3:** Meta-regression of correlations between insulin resistance and resistin.

	**Coefficient β**	**SE**	**95% CI**	***P*-value**
Insulin	0.012	0.007	(−0.004, 0.028)	0.123
Resistin	0.016	0.003	(0.008, 0.024)	0.001
Glucose	0.020	0.026	(−0.037, 0.076)	0.465
Age	−0.016	0.009	(−0.037, 0.004)	0.099
BMI	0.010	0.009	(−0.008, 0.029)	0.246

*BMI, body mass index*.

## Discussion

In the present meta-analysis, we found that resistin levels are correlated with insulin resistance in obese and T2DM patients, and this correlation was associated with resistin levels. To our knowledge, this is the first systematic review and meta-analysis using statistical methods to analyze the correlation between resistin levels and insulin resistance.

Many studies indicated that multiple adipokines, such as adiponectin and leptin were involved in the occurrence and development of insulin resistance, possibly associated with many complications (Hara et al., 2002; German et al., 2010). Resistin, an adipokine first discovered by Steppan in 2001, has been shown to induce insulin resistance in rodents. Increased resistin levels were also found in diet-induced or genetically obese mice (Steppan et al., 2001). In human studies, individuals with severe insulin resistance had higher resistin levels than individuals with normal insulin action (Zaidi and Shirwany, 2015). Therefore, we hypothesized that resistin might also play a role in insulin resistance, possibly providing new therapeutic targets for the treatment and prevention of diabetes.

Nevertheless, the relationship between resistin levels and insulin resistance has not been clarified in humans. In 2005, Al-Harithy et al. first reported a positive correlation between resistin levels and insulin resistance in diabetic women and obese or overweight non-diabetic women (Al-Harithy and Al-Ghamdi, 2005). A study in Pakistan also found a correlation in the diabetes groups and the obese non-diabetic groups (Zaidi and Shirwany, 2015). Moreover, Mabrouk et al. found a positive correlation only in obese diabetic Egyptian subjects, but not in obese non-diabetic groups (Mabrouk et al., 2013). There have also been several studies failing to find a significant correlation between resistin levels and insulin resistance in diabetic groups. Bu et al. found resistin levels had no relationship to IR in both T2DM group and normoglycemic group (Bu et al., 2012). Park et al. also observed these results in a cross-sectional study (Park et al., 2006). More and more studies concentrated on this study orientation obtained conflicting results (Mojiminiyi and Abdella, 2007; Stepien et al., 2011; Yin et al., 2012). Therefore, we attempted to reconcile these discrepancies and to draw a rational conclusion.

Among the articles searched from online databases, 15 met our selection criteria. Ten articles with Pearson's coefficients were used for meta-analysis. The overall analysis revealed a positive correlation between resistin levels and insulin resistance (r = 0.21, 95% CI: 0.06–0.35); however, heterogeneity (*I*^2^ = 59.7%, *P* = 0.003) remained significant. Consequently, we performed subgroup analysis to identify the sources of heterogeneity. According to blood resistin levels, studies were divided according to hyperresistinemia groups (*r* = 0.52, 95% CI: 0.35–0.68, *I*^2^ = 0.0%, *P* = 0.513) and normal circulating resistin groups (*r* = 0.08, 95% CI: −0.01–0.18, *I*^2^ = 0.0%, *P* = 0.455). The heterogeneity was markedly low in both group, suggesting that resistin levels were a vital influence factor in this correlation. Applying this result to all included studies, only one study did not fit (Owecki et al., 2011). This may have resulted from the high standard difference of insulin in that study, a point also mentioned by the author. We further analyzed the subgroups classified by Study design and blood sample. None reached acceptable heterogeneity.

So far, the mechanisms of insulin-resistance related effects of resistin are not clear in humans. *In vitro* studies have demonstrated that recombinant human resistin could induce insulin resistance through 5′AMP-activated protein kinase (AMPK)-dependent and AMPK-independent suppressor of cytokine signaling-3 (SOCS-3) signaling pathways in HepG2 cells (Luo et al., 2009), which is similar to the related rodent studies (Muse et al., 2004; Steppan et al., 2005). In addition, considering about the distribution of resistin in mononuclear cells in humans, inflammation might be the process which links resistin to insulin resistance. Previous studies have pointed out that resistin plays a role in pro-inflammatory processes. *In vitro* studies suggested that expression of resistin have been the result of the production of the pro-inflammatory cytokines, such as tumor necrosis factor-α (TNF-α) and interleukin-6 (IL-6) (Kaser et al., 2003; Bokarewa et al., 2005), besides, resistin could also stimulates the expression of pro-inflammatory cytokines, including TNF-α, IL-6 through the nuclear factor-κB (NF-κB, p50/p65) signaling pathway (Silswal et al., 2005). Chronic inflammation is a major and well-known cause of obesity-induced insulin resistance (Gregor and Hotamisligil, 2011), and several pro-inflammatory cytokines play an important role in the process. For example, the TNF-α signaling pathway activates intracellular kinases that inhibit insulin receptor signaling via serine phosphorylation of insulin receptor substrate 1 (IRS-1) (Hotamisligil et al., 1993), IL-6 and adiponectin also regulate insulin sensitivity (Maeda et al., 2002; Weigert et al., 2006). Resistin might exert similar functions in light of its distribution and inflammation-related functions.

In subgroup analysis, resistin levels positively correlated with insulin resistance only in people with higher resistin levels, but not in those with lower resistin levels, which was consistent with results reported by Al-Harithy and Al-Ghamdi (2005) and Mabrouk et al. (2013). The meta regression also indicated resistin levels is the source of heterogeneity. This might be explained by the hypothesis that resistin might be the principal factor which induced insulin resistance at high levels. However, the overall effect of resistin was not significant in people with low resistin levels, several other factors may dominate insulin resistance like free fatty acids, hyperosmotic stress, and TNF-α (Roden et al., 1996, 1999; Gual et al., 2003; Plomgaard et al., 2005). In the study of Park et al., people with insulin resistance did not have a correlation between resistin and insulin resistance in the case of low resistin levels, but their insulin resistance significantly correlated with TNF-α, which meet the hypothesis above (Park et al., 2006). There are also several other hypotheses regarding the controversial correlation between resistin and insulin resistance. Several single nucleotide polymorphisms (SNPs) of the resistin RETN gene have been reported to be associated with resistin concentration, while this association remains controversial in various ethnicities, possibly explaining the conflicting results of the correlation (Hivert et al., 2009). Zaidi et al. suggested that resistin causes insulin resistance when insulin reaches a certain critical level (Zaidi and Shirwany, 2015), but we did not draw this conclusion in our analysis, which may due to the limited number of relevant studies. More studies are needed to verify these hypotheses in the future.

A number of treatments targeting adipokines have recently emerged. For example, monoclonal antibody infliximab, which neutralizes TNF-α, is a new approach aimed at inflammation-associated insulin resistance (Ursini et al., 2010). That study indicated that resistin might be a therapy targeting insulin resistance in the patients with hyperresistinemia. A new therapy to reduce resistin levels may alleviate insulin resistance in the future.

Limitations in our meta-analysis should be considered. There were some studies with small sample sizes included in the present analysis, possibly explaining the heterogeneity. Few studies concentrating on the correlation between resistin and insulin resistance without reporting the correlation coefficient may have introduced bias. Some non-English articles were also excluded in our study. Therefore, larger clinical trials are needed to further validate our results.

## Conclusions

In T2DM and obese individuals, resistin levels are positively correlated with insulin resistance in people with hyperresistinemia, but not in those with normal circulating resistin levels. This study may help to suggest new targets for the prevention and treatment of insulin resistance.

## Author Contributions

KS and YL designed the program, searched and reviewed the studies, were in charge of the manuscript. YL and LS assessed the studies, extracted the data, and wrote the part of the manuscript. QL and ML extracted the data and wrote the part of the manuscript. JY, DZ, and CZ reviewed and edited the manuscript. JD directed the project, contributed to the discussion, reviewed, and edited the manuscript. JD had full access to all the data in the study and had final responsibility for the decision to submit for publication. KS and YL contributed equally to this work.

### Conflict of Interest

The authors declare that the research was conducted in the absence of any commercial or financial relationships that could be construed as a potential conflict of interest.
